# American Dental Association

**Published:** 1885-12

**Authors:** 


### AMERICAN DENTAL ASSOCIATION.

TWENTY-FIFTH ANNUAL MEETING, HELD AT MINNEAPOLIS, MINN., AUGUST 4, 5, 6, AND 7, 1885.

REPORTED FOR THE INDEPENDENT PRACTITIONER, BY "MRS. M. W. J."

(Continued from page 595.)

#### THURSDAY MORNING SESSION.

The Secretary read a letter from Dr. Geo. A. Mills, of Brooklyn, tendering his resignation. The Special Committee on the question of appropriations to the Sections, for the encouragement of original scientific research, reported through the chairman, Dr. C. N. Pierce, of Philadelphia. After referring to the scientific investigations carried on in other countries,as in Germany,under government patronage, or in England, under appropriations from the British Scientific Association and other societies, and the immense results accomplished, as compared with the meagre and incomplete results of individual effort, the prestige of the American Dental Association being compromised by lack of originality in papers and reports, the committee recommended that appropriations be made to different sections for the promotion of original investigation; the money to be expended in the purchase of materials and books, in the employment of experts in the different sciences, of artists, copyists, for expenses of printing, engraving, traveling, etc; the things that money could do in furthering this great work being almost limitless.

*Dr. W. H. Morgan*—Said that when the subject was first brought before the Association, he had announced himself in favor of the committee, but opposed to the appropriation. That since thinking the matter over, and especially while listening to the valuable paper of Dr. Ingersoll, and learning that he had traveled a thousand miles, and spent much time in pursuit of these investigations, comparing the results with those of other men, he had come to the conclusion that, though this might be a work of love with such men as Dr. Ingersoll, yet men who were able and disposed to do that kind of work ought to at least have their traveling expenses paid. He was therefore ready to vote for the appropriation.

*Drs. H. A. Smith* and *C. W. Spalding*—Objected to the insignificant amount suggested in the report, which would go but a very little way in the purchase of type-writers, microscopes, and the delicate and costly instruments required in accurate scientific investigations.

*Dr. A. W. Harlan*—Thought that such appropriations would soon require assessments, and eventually break up the Society.

NOTE.—The purchase of type-writers, microscopes and such other apparatus, is not in any way contemplated by the advocates of the resolution. If the representative Association of American Dentists is to deserve and win a reputation as a scientific body, it must foster and promote systematic, scientific investigation, and not trust entirely to desultory, individual effort.—EDITOR.

After some further discussion the report was adopted, and the sum of two hundred dollars each appropriated to Sections V, VI, and VII.

*Dr. E. Parmly Brown*—Wished to present a crown which he had just perfected in connection with bridge-work. The most crowns were weakened by being too much hollowed out; the strain on a tooth was not equal, the lateral strain being insignificant as compared with the strain in biting. He had consequently invented a broad, flattened pin, which gave added strength where the leverage was greatest. His crowns were also rapidly inserted. He had put in three between nine and one o'clock, for a lady who was a professor of vocal music, and could only give him those four hours for the entire operation.

*Dr. C. S. Stockton*—Said there was no subject now engaging the attention of the profession so much as that of bridge-work. That it pertained as closely to mechanical as to operative dentistry, and that he had hoped it would have been given to the former section. As it had been included under operative dentistry, he had a case he would like to report. There were in the mouth the six front teeth, one right bicuspid, and two wisdom teeth in the lower jaw. In the upper jaw were the six front teeth and two wisdom teeth. The six upper front teeth were entirely covered and hidden by the lower incisors in occlusion, presenting a very unsightly appearance. Several attempts had been made to supply artificial dentures, without success. He had placed gold caps over the wisdom teeth, throwing the jaws apart so as to leave the upper incisors only slightly behind the lower, filling in the spaces with bridge-work. The operation was thoroughly satisfactory, the patient pronouncing it a blessing that was beyond price.

*Dr. E. Parmly Brown*—Said that the Herbst method of filling might be good, but it was not the best. It was going back to old styles, and necessitated the cutting away of good material in order to make simple cavities. No man would dare to say that the Marshall H. Webb method of preparing cavities was not correct.

*Dr. J. D. Patterson*—Said he thought the Herbst method would prove unreliable. He had experimented with it for two years, both at the bench and in the mouth. He could fill the same cavities more rapidly and more thoroughly with annealed soft gold or cylinders. He thought the fillings made at the clinic yesterday would fail within six months.

*Dr. G. C. Daboll*—Said that the best point in the Herbst method was the use of the matrix. This insures ease and certainty, and saves time and labor. It makes a simple crown-cavity of the most difficult posterior proximal cavities in molars and bicuspids.

*Dr. W. A. Spalding*—Said that the Herbst method was good in some places, and in some cavities, but he noticed that its most enthusiastic advocates finished their work with the electric mallet. The one point was to put the gold in solid, no matter by what method, whether Herbst's, or Smith's, or Brown's. Do what is best for the case in hand. One says he *always* uses the mallet; another *always* uses the matrix; but there are cases where neither is available. Each man should use his own method, and exercise his own judgment.

*Dr. W. C. Barrett*—Said that it was not individual cases, but basal laws that should be discussed. There were three principles involved in filling teeth with gold. One was the wedging process, in use forty years ago, requiring non-cohesive gold, one piece sliding past the other, each held in place by the mechanical friction of the wedge. By this system good work was done in saving teeth. Then came cohesive gold, impacted by blows, starting from a single point where it was securely anchored, and built up piece by piece, making a solid, homogeneous mass, restoring nature's contour. These two methods involved diametrically opposite principles. In one the gold surfaces must slide upon each other; in the other they must not. In one it was wedging; in the other it was welding.

The Herbst method is entirely different in principle; the gold is burnished down; but each of the three methods has its advantages and its merits, and each has something lacking. By the first method tooth form cannot be restored; by the second, gold is not brought in such absolute contact with a smooth wall. To produce absolute contact the gold must not be impacted or driven with sharp points; it must be spread out and worked down smoothly.

The Herbst system of rotary burnishing produces the most perfect contact with the walls of the cavity, but the building up of a solid, homogeneous mass, requires the mallet.

To produce finished, complete work, the mastery of all these systems is required. Use the Herbst method, in those cases in which it is applicable, to insure contact with the walls of the cavity; use the mallet to build up the mass and condense the surface, then you will have a perfect operation. The operator who excludes all but his own way—who says my way is always the right way, and the only right way, and all others are always wrong—will not succeed, will not make a good teacher. We must select that which is the best adapted to each individual case.

*Dr. E. Parmly Brown*—Said that wherever gold was put in human teeth, and needed there, it was needed absolutely solid. He did not believe the Herbst, or rotary process, would put gold in any angle or pit, and he had challenged Herbst to meet him, either in Europe or in America He had been asked to fill gold tubes as a test, but that would not prove anything, and that was not his business. Gold could doubtless be worked against a smooth glass surface with the rotary motion, but that was no proof of what it would do, in a cavity, in preserving the tooth.

*Dr. M. L. Rhein*—Said that Dr. Barrett had struck the keynote in regard to combining different methods according to the case in hand. The proper kind of gold, the peculiar German gold with which to work on the German plan, had only been very recently introduced into this country. The Herbst method could only be successfully employed with that gold which was made especially for that process—a very soft, non-cohesive gold, which was like working the burnisher in a roll of butter—absolutely non-cohesive in the bottle, although as solid in the tooth as if malleted. He did not consider his clinic a fair test, as he had worked with borrowed instruments. Up to the point where he used the mallet, none of his gold had been annealed, but the best operators had examined it and pronounced it thoroughly welded. The failure of work done two or three years ago, claimed as done by the Herbst method, was due, not to the method, but to the use of ordinary gold. Work that he did last September, by the Herbst method, with the German gold, was as perfect as if done yesterday. He had been a pupil of Dr. Webb in the method of preparing a cavity and contouring, but for the Herbst method no retaining pits or painful undercuts were required, this being one of its great advantages. For finishing the surface, and for contouring, he used the mallet, combining the two methods, annealing the gold very slightly for the mallet. He said that Herbst had shown the greatest ingenuity in the adaptation of the matrix in the anterior teeth, making proximal cavities as simple as molar crown cavities. It was just as necessary to have good, strong walls, as if all the work was to be malleted. In very large cavities the walls could be lined with gold by the Herbst method, the centre filled in with oxy-phosphate, in which gold is again anchored and malleted for the masticating surface. There is not the slightest necessity for impacting a large amount of gold, if the enamel edges are perfectly sealed and leakage from the exterior prevented. The quantity of gold impacted is not a material point, so that the tooth is preserved.

*Dr. W. A. Spalding*—Said that he understood the Herbst method to mean the introduction of a new gold, a very pure gold, and only that kind of gold. He used another manufacture of gold, and had laid one piece on another without annealing, and burnished them together so that they could not be picked apart. Any kind of gold that would cohere solidly was good gold, whether the work was done by the Herbst method or by any other method; with a peculiar kind of gold or with any other kind of gold. The point was simply to have all moisture excluded. It was narrow-minded to feel restricted to the use of any one method, or any one material.

*Dr. L. C. Ingersoll*—Said there were two or three principles involved. The surfaces brought into contact by burnishing were held together by atmospheric pressure. When they adhered from the effect of rapid rotary pressure, it was from electrical attraction, which developed cohesion. By this means, taking advantage of these two principles, Dr. Herbst himself can restore contour by his system.

*Dr. W. H. Atkinson*—I am using a manufacture of gold upon platinum and iridium. This foil makes a harder surface for mastication than any other known, comparing with ordinary soft gold as steel with soft iron. It can be used in Nos. 120 or 60 or 30. Its great advantage is its hardness; also its near approach to tooth color. It adheres to ordinary gold better than to tin. Platinum and iridium, with a large proportion of 120 gold foil, makes excellent plate for clasps, when rolled to 24 American gauge. To make a uniform plate, flow melted gold scraps on the platinum and roll. If heated it will make nodules. Lightly hammer these together and run them through the mill. Anneal in filling, and use hot from the lamp. This gold and platinum is in cylinders and in foil, and makes a soft gold of good color. The addition of iridium gives a good, hard masticating surface.

*Dr. E. T. Darby*—Would ask Dr. Atkinson if he uses 60 and 120 foil as a rule?

*Dr. Atkinson*—I don't care whether it is 60 or 120 or 420! I cut it into strips, anneal, and carry home.

*Dr. W. W. Allport*—While others are speaking, many things pass through my mind that might be useful to younger members. In listening to the discussions regarding the different methods of using gold, and different manners of operating, I am reminded of the saying of an eminent Divine: "There is no heresy so false that it has not in it a germ of truth." It is equally the fact that there is no doctrine so true that it has not in it something false. One speaker insists that a hard and solid filling is absolutely essential. If a filling is so packed against the wall of the tooth as to resist mastication and exclude moisture, that is all that is necessary. Going beyond that is going beyond the point of utility. The additional hardness is a damage to the tooth, being unyielding to thermal changes. At the meeting of the Association in Boston, I called attention to the difference between cohesion and non-cohesion. Non-cohesive gold simply slides on the surface without clogging. No man here will say that the old-fashioned operators did not save as large a proportion of the teeth they filled as are saved in proportion to-day, or that any more beautiful operations are made now than were made then. Filling and saving a tooth is one thing—building up a tooth is another. There is nothing better for filling and saving teeth than non-cohesive gold. As I said a year ago, I can see much good and much evil in the Herbst method. It offers much that is attractive; in some ways it may be good, but not for practical every-day work, and I predict great evil if it is generally adopted. I judge from what I have heard, and from what I have seen—I have not tried it myself. I do not dispute that it may be burnished against the walls, but not into undercuts. How are you going to use straight instruments in uneven cavities? You must cut away and destroy tooth-substance. No man has the right to destroy where he can save. The new gold may be the best ever seen, but the method of using it is not good; that is, in my opinion. If you employ the Herbst method at all, use it in open cavities, of easy access. If I were to use it I should reverse the order and fill the cavity with the mallet, and finish with the burnisher, and for this I would use Abbott's bulbs.

*Dr. G. R. Thomas*—We all seek the same end by different roads. There are thorns and thistles which are easy to uproot if handled in the right way, but some men cling to their own narrow conceptions. There is a great difference between making beautiful fillings and being a good dentist. Fillings may be beautiful to the eye and yet not save the teeth. When one method is used exclusively, there is danger of carrying it too far. It is due to the patient to produce good results. It must be remembered that we are dealing with a frail, sensitive structure. There is more danger to the teeth from over-operating than from not enough. Young men are urged to use the matrix on all occasions, but good results can be produced without it. All students were once taught to use vulcanite rubber, and now few can use anything else. Learn first to make good gold fillings without a matrix, otherwise there will be trouble in cases where a matrix cannot be used.

*Dr. H. J. McKellops*—Said he had listened attentively to Drs. Allport and Thomas. He would ask Dr. Allport what he called adhesive and non-adhesive? What he used the lamp for when using the mallet? He had noticed that in employing soft foil, Dr. Allport took a No. 4 roll in his fingers and thrust it in the flame to make the point stick. Did not that make it adhesive? He desired men to practice what they preach. Concerning the matrix, there were no instruments more useful than those of Dr. Jack; most essential where it was important to save time, or where we cannot get up to the cervical wall. Young men must be taught to use it in the places where it would accomplish good work. Referring to what Dr. Atkinson had said of iridium-gold, Dr. McKellops said that he had not had as much experience with it as with platinum-gold, but that which he had was not satisfactory. It was too stiff. It would not yield, or work under the lamp as he wished. He got better results with No. 60 platinum-gold rolled. He had found nothing equal to it for the masticating surface. The first he had seen was from Blake, of California, in 1876. He had been using it ever since. He could almost produce any shade he wanted for contouring front teeth; across the table it would hardly be observed that the teeth were filled. He used the shades 1, 2, and 3, and nothing less than No. 60.

(TO BE CONTINUED.)

### AMERICAN DENTAL SOCIETY OF EUROPE.

### THIRTEENTH ANNUAL MEETING, HELD IN BERLIN, AUGUST, 1885.

REPORTED EXPRESSLY FOR THE INDEPENDENT PRACTITIONER.

(Continued from page 605.)

### SEQUELÆ OF CARIES OF TEETH.

Dr. Gottinger read the following paper upon this subject:

In studying disease it is customary to discuss, besides the morbid phenomena of the process and the resulting structural changes in the part affected directly, known as primary lesions, such other changes as are produced secondarily, or in parts not directly involved. These indirect consequences of a disease are known under the name of sequelæ. A distinction is to be made between the sequelæ of a disease and its complications. The latter term signifies an intercurrent disease, arising during the presence of another, and not necessarily nor directly produced by the former, whilst sequelæ are directly dependent on the pre-existing disease. The study of the sequelæ of a disease is hardly less important than its primary consequences, for, from the intimate anatomical and functional relation, which the single constituents of the economy entertain to the entity, it seems that even lesions of a purely local nature always influence the entire economy; nay, that a lesion to even a single cell cannot exist without producing an impression on the whole organism. As a rule, however, continuity of structure, or the connection by the blood or nervous channels, determines this involvement. We propose to limit this discussion to the sequelæ produced by caries of the teeth. We may conveniently consider them under three categories, as produced:—

First, by proximity of structure.

Second, by reflex nervous action.

Third, by communication through the circulatory system. Disorders of the teeth may be transmitted to all the structures of adjacent parts with which they entertain relations, either of continuity or contiguity.

The first and most frequent involvement in disorders of teeth, is probably that of the saliva, and recollecting the important function of saliva in digestion, we will readily appreciate the importance of its integrity and the mischief produced by its morbid alteration.

Besides, the saliva reabsorbed by the blood will impart its own taint to it. Next to the saliva, the air entering the respiratory tract also undergoes certain changes when the teeth are diseased. This change, of course, will not be very marked, owing to the brief, momentary contact of the air with the diseased structures. But, however slight such an alteration may be during a single inspiratory act, the effect must become cumulative by the frequency of those acts.

Next in order to be affected are manifestly the gums, due to their intimate anatomical connections. I think that any decided morbid alteration of the teeth, especially those of a temporary and inflammatory nature, always involves the gums. Besides, the great vascularity of these structures predisposes to such an affection. That even the tongue cannot retain its normality during the periods of dental disorders, seems likewise quite natural. It may be said briefly, that all the surrounding mucous structures suffer more or less, the epithelial cells and mucous glands probably being first attacked. Profound lesions of the teeth, especially caries, not infrequently travel down the maxillary bones, attacking the periosteum and the bony structures themselves. This is more apt to be the case in periodical affections of the teeth, where the diminished vascularity of the system creates a predisposition to such lesions. Even comparatively remote structures, such as the laryngeal or digestive tract, may be affected. In this case, the involvement is said to take place by contiguity of the mucous membrane, As a rule, the extension to the respiratory tract will be found limited, the beginning of the ciliated epithelium forming the line of demarcation, as diseases in these mucous membranes rarely travel beyond the limits of their own epithelium. The circulatory system forms an important and dangerous channel for the communication of affections of the teeth ; important, because all products of morbid processes in the economy, unless they be discharged externally or become organized *in loco*, are carried off by the circulation; dangerous, because the greatest lesions of the economy, probably, are those produced by the presence of septic material in the blood.

We now proceed to sequelæ of altered teeth, transmitted by nervous influences. We will begin at once with an illustration. The fur of the tongue, although often produced by an altered condition of the blood in febrile affections, or by local affection of the mucous membrane, does not infrequently result from the presence of diseased teeth. Every attentive examiner of clinical patients must have noticed the peculiar furry condition of the tongue in case of decayed molars, mostly unilateral, on the side of the affected molar. This condition seems to be produced, less by the contiguity of the structures than by reflex action, involving primarily the second and third division of the trifacial nerve, this nerve presiding over nutrition and repair of the parts. That it is really a reflex action, appears from the fact that the same furry condition of the tongue is produced occasionally by intercranial disease, or by fracture of the base of the skull. A case of tubercular deposit on the Gasserion ganglion, and another where fracture, running through the foramen rotundum, produced the mentioned unilaterally furred tongue, were observed by John Hilton. But another explanation of unilateral fur in presence of a diseased tooth seems equally plausible, namely, that the half of the jaw containing the diseased tooth is far less used in mastication than the other, and hence no removal of the epithelium of one-half of the tongue takes place by friction of the food. The same unilateral fur was also observed by Hilton, in case of inflamed tonsils. In this case the nervous connection related to the glosso-pharyngeal nerve, which is distributed to the tongue, and also sends branches to the tonsils. A more curious instance of nervous transmission from the teeth is the appearance of gray hair on the temple, dependent on a decayed molar on the same side. A case is reported in which a patient with the affection mentioned applied for treatment at Guy's Hospital, London. An examination revealed the presence of a decayed molar in the left lower jaw. The extraction of the tooth brought with it a prompt return to the normal conditions. In this case the auriculo-temporal branch of the third division of the trifacial probably caused the stated affection.

Even the ear may, by reflex action, become the seat of disorders, when the teeth are affected. This is illustrated by the case of Dr. Addison, of London. This gentleman applied to the most eminent specialists of the ear for treatment for an ulceration of the auditory canal. The remedies given failed to benefit him; but an extraction of a diseased molar in the lower jaw on the same side proved a quick and complete relief. A few highly interesting cases bearing upon our discussion are reported by H. Hancock, surgeon to the Charing Cross Hospital, London.

DISCUSSION.

*Dr. Miller*—The subject which has just been discussed is one of the most interesting and important that we dentists have to deal with; it is also this aspect of oral diseases which entitles them to the most serious consideration of the pathologist and general practitioner. A very valuable contribution to this subject has also lately been made by a medical man, Dr. Von Kaezorowski, of Posen. He directs especial attention to the fact that loss of appetite, nausea, vomiting, etc., may be, and often are, the result of a diseased condition of the mucous membrane of the mouth produced by caries of the teeth. He refers to cases where food, when administered by the pump, was perfectly well tolerated by the stomach, but when the patient attempted to take food in the ordinary way, vomiting was produced the minute it came in contact with the fauces. A thorough disinfection of the oral cavity and fauces was followed by return of the appetite, disappearance of the nausea, and restoration to health.

(TO BE CONTINUED.)

